# Preparation and Performance of Ceramic Tiles with Steel Slag and Waste Clay Bricks

**DOI:** 10.3390/ma17081755

**Published:** 2024-04-11

**Authors:** Ying Ji, Enyao Li, Gang Zhu, Ruiqi Wang, Qianqian Sha

**Affiliations:** 1 College of Materials Science and Engineering, Xi’an University of Architecture and Technology, Xi’an 710055, China; lienyao@xauat.edu.cn (E.L.); 13363941368@163.com (R.W.); shaqianqian@xauat.edu.cn (Q.S.); 2 China Building Materials Industry Construction Xi’an Engineering Co., Ltd., Xi’an 710065, China; cbmxe2023@163.com

**Keywords:** ceramic tiles, steel slag, waste clay bricks, solid waste resource utilization

## Abstract

Steel slag and waste clay bricks are two prevalent solid waste materials generated during industrial production. The complex chemical compositions of these materials present challenges to their utilization in conventional alumina silicate ceramics manufacturing. A new type of ceramic tile, which utilizes steel slag and waste clay brick as raw materials, has been successfully developed in order to effectively utilize these solid wastes. The optimal composition of the ceramic material was determined through orthogonal experimentation, during which the effects of the sample molding pressure, the soaking time, and the sintering temperature on the ceramic properties were studied. The results show that the optimal ceramic tile formula was 45% steel slag, 35% waste clay bricks, and 25% talc. The optimal process parameters for this composition included a molding pressure of 25 MPa, a sintering temperature of 1190 °C, and a soaking time of 60 min. The prepared ceramic tile samples had compositions in which solid waste accounted for more than 76% of the total material. Additionally, they possessed a modulus of rupture of more than 73.2 MPa and a corresponding water absorption rate of less than 0.05%.

## 1. Introduction

**Figure 1:**
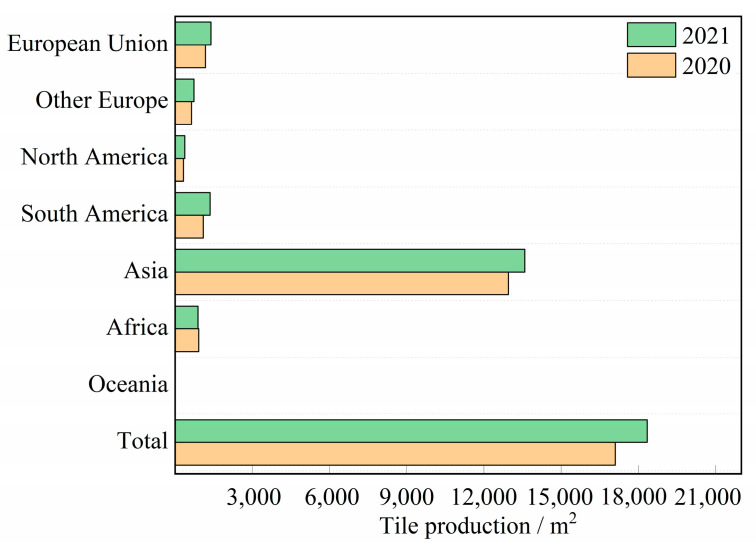
Global ceramic tile production.

In recent years, global ceramic tile production and consumption have rapidly recovered from the declines caused by the new coronavirus outbreak. According to the data presented in Figure 1 [1], global ceramic tile production reached 18,339 million square meters in 2021, representing a significant increase of 7.2% from the previous year’s production. Except for Africa, all the regions depicted in Figure 1 (including Asia, Europe, North America, and South America) witnessed some degree of growth. It is particularly noteworthy that Asia emerged as the foremost tile-producing region worldwide; it generated a total output of 13.585 billion square meters, which accounted for approximately 74% of the global production. Simultaneously, the building ceramics industry has witnessed an increase in the consumption of raw materials, such as clay, quartz, feldspar, and quartz sand, to accommodate its expanding production capacity. However, recent studies have shown that utilizing low-cost industrial solid waste as substitutes for traditional raw materials or as additives to enhance the performance of ceramic materials can effectively alleviate resource scarcity issues and further mitigate the continuously increasing production costs [2,3,4,5,6]. It is noteworthy that, because of its substantial and expanding production capacity, the building ceramics industry has significant potential to mitigate the large-scale accumulation of solid waste [7]. Consequently, this approach can not only resolve the problem of solid waste accumulation but can also mitigate the associated production costs within the ceramics industry, thus contributing toward the sustainable development of the building ceramics sector.

Steel slag and waste clay bricks are two common types of solid waste. However, due to the absence of effective treatment methods, there has been a substantial buildup of these materials, resulting in serious environmental problems [8,9,10]. In recent years, steel slag has been found to be valuable as a coarse aggregate or a mixing material in concrete, cement, backfilling materials, and road construction materials [11,12,13]. Furthermore, it can enhance soil quality and fertilizer production in agricultural practices [14,15,16]. Due to its high alkalinity, steel slag can also serve as carbon capture and storage to mitigate greenhouse gas emissions [17,18,19,20]. Additionally, steel slag exhibits adsorption properties that enable the removal of pollutants in wastewater treatment processes [21,22,23,24]. Simultaneously, it can be utilized as a raw material for ceramic manufacturing to enhance the performance and durability of ceramic products [25,26,27]. Recent research regarding recycling and the reuse of waste clay bricks has primarily focused on building materials [28,29,30,31,32]. However, the combined utilization of steel slag and waste clay bricks remains limited despite these treatments.

In recent years, scholars have discovered that steel slag is predominantly composed of silicate minerals and solid solutions formed by bivalent metal oxides such as FeO, MgO, and MnO, while waste clay brick primarily consists of quartz and andesine. Based on their compositional characteristics, these materials can be used to manufacture ceramic products [33]. However, the traditional SiO_2_-Al_2_O_3_-K_2_O system may not be suitable for steel slag ceramics because it requires limited amounts of Fe_2_O_3_ and CaO (the Fe_2_O_3_ content must be less than 0.8 wt.% and the CaO content must be less than 3 wt.%). Zhao et al. [34] proposed a new pyroxene ceramic system, CaO-MgO-SiO_2_-Al_2_O_3_-Fe_2_O_3_-K_2_O (Na_2_O), that is different from the traditional SiO_2_-Al_2_O_3_-K_2_O (Na_2_O) ternary system. The ceramic products produced by this system have good mechanical properties and flexural strengths that can reach 143 MPa; additionally, their crystal phases are all pyroxene-group minerals. Because of the different steel fabrication process regimes adopted by various steel plants, steel slag composition is highly variable. In addition, Zhao et al. [35] further classified the ceramics formulations into (a)-series CaO-Al_2_O_3_-SiO_2_ and (b)-series CaO-MgO-SiO_2_ systems based on the Al_2_O_3_ and MgO contents. The primary crystalline phases in the (a)-series samples included calcium feldspar, α-quartz, and magnetite, while the main crystalline phases in the (b)-series samples were pyroxene, tremolite, or a pyroxene solid solution; all these represented de novo crystalline phases. Notably, the flexural strengths for the (a)-series and (b)-series samples were 53.47 MPa and 99.84 MPa, respectively (which were both greater than the 35 MPa strength proposed by the Chinese national standard). Liu et al. [36] successfully synthesized a building ceramic material that complied with international standards by utilizing 60~70% red mud, 20~30% steel slag, and 10% talcum powder, followed by sintering at a temperature of 1170 °C. Tabit et al. [37] incorporated steel slag into fly ash at rates of 10–50 wt.%, which resulted in the successful synthesis of calcined feldspar-based ceramics with optimal properties. They were fabricated with a CaO–SiO_2_ ratio of 0.46 and a sintering temperature of 1188 °C. The calcium feldspathic ceramics prepared using this method exhibited a low thermal conductivity (0.39 W/m-K), a high dielectric constant (6.03 at 1 MHz), and a compressive strength of 41 MPa; these properties made the ceramics promising candidates for insulation applications. The standard full factorial design (GFFD) method was employed by Teo et al. [38] to optimize the sintering temperature and composition formula (body) of a clay ceramic material that incorporated electric arc furnace slag. Their findings demonstrated that adding 50 wt.% of electric furnace slag and sintering at 1180 °C yielded maximum bending strength, minimum moisture absorption, and minimum apparent porosity values. Mymrin et al. [39] used printed circuit boards, red mud from bauxite processing, and steel slag to completely replace the clay and quartz raw materials while preparing ceramic materials. The resultant flexural strength was 15.39 MPa, and the water absorption was between 4.47 and 38.45%. Ceramic foam is a highly appealing material that has extensive applications in diverse fields. The utilization of steel slag in the production of ceramic foam offers an alternative solution. Xu et al. [40] used steel slag, kaolin, feldspar, and quartz as the primary raw materials while producing a new ceramic foam material. They also incorporated SiC as a high-temperature foaming agent. With a steel slag content of 40 wt.% and a SiC particle diameter of 20 μm, the resulting material exhibited a water absorption rate of 2.59%, a total porosity of 55.91%, a bulk density of 1.33 g/cm^3^, and a compressive strength of 1.21 MPa. Tang et al. [41] discovered that a raw material with 94 wt.% of sand shale and 6 wt.% of steel slag could be utilized to fabricate a foam ceramic material with a remarkable strength of 29.98 MPa, a bulk density of 0.821 g/cm^3^, an overall porosity of 67.22%, and a closed porosity of 55.27%. Previous studies mainly concentrated on ceramics that incorporate pyroxene or plagioclase as the primary crystalline phase. Akermanite is mainly used in medical bioceramics [42,43], but its potential as the primary crystalline phase in ceramic tiles has been overlooked, limiting its applications for other forms of solid waste utilization. Furthermore, despite the similarities between the clay compositions of discarded clay bricks and traditional ceramic materials, the potential for using waste clay bricks to produce recycled ceramics remains largely unexplored.

The primary objective of this research was to employ steel slag and waste clay bricks as raw materials to produce ceramic tiles that conform to quality standards, thereby offering a novel resource utilization method for these two types of solid waste. In this study, ceramic tiles, in which akermanite was the main crystalline phase, were prepared using steel slag and waste clay bricks as the primary raw materials. An orthogonal experimental technique determined their compositions. They were fabricated using traditional ceramic processing methods, and their physical and mechanical properties and microstructures were investigated. In addition, the effects of process parameter variations on the microstructures, crystalline phase transitions, and physical properties of the ceramics were also investigated. This study can also provide a reference for using other calcium-rich waste materials.

## 2. Materials and Methods

### 2.1. Materials

**Figure 2:**
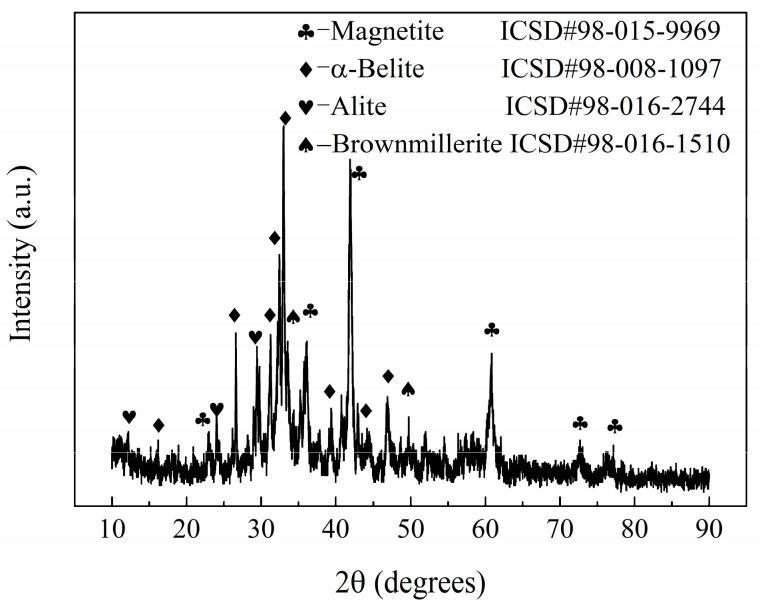
XRD results for the steel slag.

**Figure 3:**
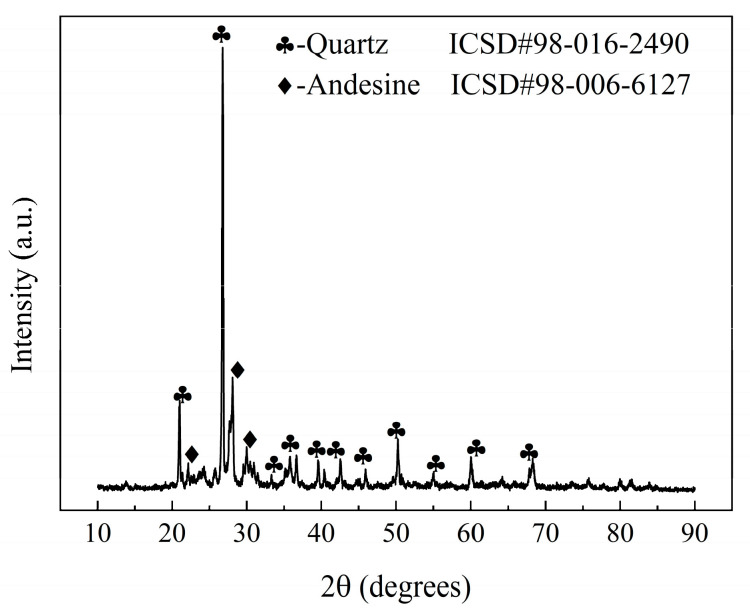
XRD results for the waste clay brick powder.

The raw materials used in this study include converter SS (manufactured by the Longgang Group, located in Xi’an, China), waste clay bricks (manufactured by the Qingcaigou Construction Waste Disposal Plant, located in Xi’an, China), and talcum powder (manufactured by the Guiguang Talcum Development Co., Ltd. located in Guilin, China). The chemical and phase compositions of the powdered materials were analyzed using X-ray fluorescence (XRF) spectrometry and X-ray diffraction (XRD). The results are presented in Table 1 and Figure 2 and Figure 3.

The steel slag consisted of several elements, including CaO, Fe_2_O_3_, SiO_2_, MgO, and Al_2_O_3_, and it had a complex physical composition. Its crystal phase composition, in addition to dicalcium silicate and tricalcium silicate, also included other substances, such as magnetite and brownmillerite. The waste clay brick powder contained SiO_2_, Al_2_O_3_, Fe_2_O_3_, CaO, MgO, Na_2_O, K_2_O, and TiO_2_, and other alkali metal oxides. Its crystal phase composition was simpler, as it was primarily composed of quartz and andesine. Waste clay bricks can replace clay and feldspar as raw materials for ceramics. The talc was mainly composed of SiO_2_ and MgO.

### 2.2. Orthogonal Experimental Design

In this study, steel slag and waste clay bricks were used as the primary raw materials in the preparation of architectural ceramic tiles, while talc was used as an auxiliary raw material. To investigate the effects of the steel slag content, the waste clay brick powder content, the sintering temperature on the water absorption rate, and the modulus of rupture of the ceramic tiles, an L_9_ (3^3^) orthogonal experiment was designed. The three factors in the orthogonal experiment were the steel slag content, the waste clay brick content, and the sintering temperature. Three levels were selected for each factor, and the values that corresponded to each level of each factor are presented in Table 2. The orthogonal experiment design is shown in Table 3. The amount of talc used in the experiments remained fixed at 25%, the molding pressure was maintained at 20 MPa, and the soaking time was held at 90 min. After conducting comprehensive testing on each group of formulations listed in Table 3, a range analysis was performed to identify two groups of optimized formulations, which are presented in Table 4. These optimizations were based on the minimum water absorption rate and the maximum modulus of rupture. Control experiments were conducted for these two groups of formulations, which ultimately resulted in a selection of the optimal group with the best overall performance. Subsequently, single-factor experiments were conducted to analyze the effects of other parameters, including the molding pressure, the sintering temperature, and the soaking time, on the performance of the ceramic tiles during production.

### 2.3. Single Factor Experiment of Process Parameters

After obtaining the optimal formula through range analysis of orthogonal experimental results, additional single-factor experiments were conducted on key variables such as molding pressure, sintering temperature, and holding time in order to further investigate the optimal process parameters for ceramic tile production.

Firstly, single-factor experiments were conducted using the optimized formulation obtained from orthogonal experiments under the conditions of a sintering temperature of 1190 °C and a holding time of 90 min. The influence of molding pressure ranging from 15 to 30 MPa on the water absorption rate and modulus of rupture of ceramic tiles was investigated. The optimal molding pressure was determined based on selecting the group with the lowest water absorption rate and highest modulus of rupture. The experimental process parameters are presented in Table 5, where each group’s formulation was derived from the optimized formulation obtained through orthogonal experiments.

Secondly, the optimal molding pressure determined in the previous step was employed, and a soaking time of 90 min was used to conduct a single-factor experiment within the temperature range of 1185–1200 °C for sintering. The optimum sintering temperature was identified as the one corresponding to the group exhibiting the lowest water absorption rate and highest modulus of rupture. Table 6 presents the experimental process parameters, where each group’s formulations were based on the optimized formulations obtained from the orthogonal experiments.

Finally, a single-factor experiment was conducted to vary the soaking times (ranging from 30 to 90 min) under the optimal molding pressure and sintering temperature determined in the previous steps. The soaking time corresponding to the group that exhibited the lowest water absorption rate and highest modulus of rupture was selected as the optimal duration for soaking time. The experimental process parameters are presented in Table 7, and the formulations of each group were based on the optimized formulations obtained from the orthogonal experiments.

By conducting the aforementioned single-factor experiment, we can comprehensively investigate the impact of these three process parameters on the performance of ceramic tiles and determine the optimal process parameters based on the refined formulation.

### 2.4. Raw Material Pretreatment

**Figure 4:**
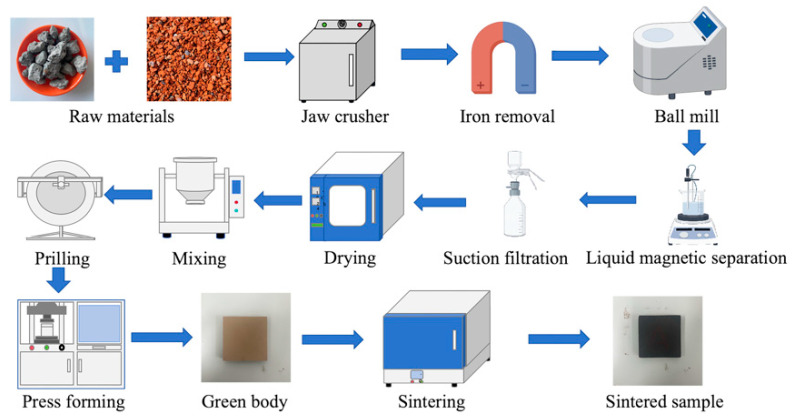
Schematic diagram of the sintered tile preparation process.

The ceramic tile preparation method, which is illustrated in Figure 4, involved multiple stages. During the initial sample pretreatment stage, the steel slag was crushed into fragments with diameters of 1–2 cm using a jaw crusher. Then, a strong magnet was employed to remove any remaining iron filings and Fe_3_O_4_ components. The de-ironed steel slag was ground for two hours in a ball mill with dimensions of 500 mm × 500 mm that rotated at a speed of 48 rev/min. Subsequently, it was sieved through a screen with openings of 75 μm. The ground steel slag was then weighed, poured into a beaker containing anhydrous ethanol, and placed in a magnetic stirrer. The stirring speed was controlled within 15–20 rev/s due to the differences between the densities and specific magnetizations of the steel slag components. In the ethanol solution, the minerals with low densities and weak magnetizations were suspended in the upper layer, while those with high densities and high magnetizations were situated in the lower layer. The solid–liquid mixture was taken from the upper and lower layers, filtered, and dried in an oven at 105 °C for future use. The waste clay bricks were cleaned to remove sludge from their surfaces, then they were crushed by a jaw crusher into fragments with diameters of 1–2 cm. They were then placed into a ball mill with dimensions of 500 mm × 500 mm and ground for two hours at a rotational speed of 48 rev/min. The ground waste clay bricks were filtered through a 75 μm sieve and dried in an oven at 105 °C for future use.

### 2.5. Sample Preparation

After the raw materials were pretreated, the preparation of the tile samples commenced. The samples’ constituents included steel slag, waste clay brick powder, and talcum powder, which were precisely weighed according to the mass percentages specified in Table 3 so that the total component mass was 500 g. The raw materials were then thoroughly mixed in a mixer for 60 min before being placed in a disc granulator at a speed of 40 rev/min. Water was carefully added to the coarse material using spray cans to ensure that the manufactured powder had a water content between 7% and 9%. The finished rough material was then strained, and a fine material with particulate diameters between 0.85 mm and 0.178 mm was obtained. The fine material was then encapsulated in a sealed bag and placed in a cool location for 24 h so the moisture could be distributed evenly. Afterward, the aged material was poured into 50 mm × 50 mm metal molds and subjected to mechanical pressure to form dense samples with dimensions of 50 mm × 50 mm × 8 mm. A molding pressure of 20 MPa was used, along with a loading rate of 0.5 kN/s. The green bodies were then dried in an oven at 105 °C for 24 h so they would obtain a particular strength. Finally, the green bodies were placed into a mullite container, which was placed into a high-temperature furnace. Atmospheric pressure sintering was then performed according to the designed firing regime, which resulted in the final ceramic tile samples.

### 2.6. Sample Performance Measurements

#### 2.6.1. Macroscopic Performance Measurements

The water absorption rate and the modulus of rupture are critical parameters used to evaluate ceramic materials. International standards and specifications for ceramic tiles typically mandate specific requirements for the values of these properties to ensure compliance with quality standards. The water absorption rate provides information regarding the tile density and pore structure. A low rate signifies that a tile has high density and strength; conversely, ceramics with high water absorption rates are prone to expansion, cracking, or breakage when exposed to temperature fluctuations, humidity changes, or chemical erosion. The modulus of rupture reflects the structural strength of the tile material by indicating its ability to resist external forces without breaking.

The vacuum method outlined in the ISO 10545-3 standard [44] was used to measure the water absorption of the ceramic tiles. The data obtained in this experiment represented the mean values of 10 samples for each set of formulations. First, the mass of a dry tile was measured, with an accuracy of 0.01 g, and recorded as *m*_1_. The tile was then placed vertically into a vacuum chamber without contact with other tiles. The chamber was vacuumed to 10 ± 1 kPa, and this vacuum pressure was maintained for 30 min. Next, enough water was added so the tile was submerged to a depth of 5 cm, and the tile was left to soak for 15 min. The tile was then removed from the water, and a piece of soaked chamois leather was used to gently dry its surface. The mass of the water-impregnated tile was then immediately measured with the same accuracy as was used for the dry tile, and this mass was recorded as *m*_2_. Finally, the water absorption, *E*, was calculated using Equation (1):

(1) $$E=\frac{m_{2}-m_{1}}{m_{1}}\times 100\%$$

Reckon the apparent volume *V* (cm^3^) by employing Equation (2).

(2) $$V=m_{2}-m_{3}$$

where *m*_3_ represents the mass of a brick suspended in water after being saturated with water using the vacuum method.

By utilizing Equation (3), the volumes of the open pore part *V*_0_ can be calculated.

(3) $$V_{0}=m_{2}-m_{1}$$

The apparent porosity *P* is quantified as the percentage of the open pore volume relative to the apparent volume of the sample and can be determined using Equation (4).

(4) $$P=\frac{V_{0}}{V}\times 100\%$$

The modulus of rupture was determined using the three-point flexural method [45]. A sample was placed on a flexural fixture with the center bar equidistant from the two supporting bars. The sample was uniformly loaded at a rate of 1 N/mm^2^·s ± 0.2 N/mm^2^·s, and the rupture load, *F*, was documented. The modulus of rupture, *R*, was then computed using Equation (5):

(5) $$R=\frac{3Fl_{2}}{2bh^{2}}$$

where *F* represents the rupture load, in N; *l*_2_ is the distance between the two support bars, in mm; *b* is the specimen width, in mm; and *h* denotes the minimum thickness of the sample fracture surface as measured along the fracture edge after the test, in mm.

Linear shrinkage (*Ls*) is determined by the difference in length between the green body (*L*_1_) and the sintered sample (*L*_2_). Please refer to Equation (6) for the calculation formula.

(6) $$Ls=\frac{L_{1}-L_{1}}{L_{1}}\times 100\%$$

The calculation formula for the bulk density *B* is presented in Equation (7).

(7) $$B=\frac{m_{1}}{V}$$

#### 2.6.2. Microscopic Performance Measurements

The phase compositions of the optimized samples were determined using a D/MAX2000 X-ray diffractometer (Rigaku, Tokyo, Japan) with a Cu target, a tube voltage of 40 kV, a tube current of 26 mA, a power of 1600 W, a scanning angle in the 10–90° range, a step size of 0.02, and a scanning speed of 5°/min. The pore structure, densification degree, crystal morphology, and elemental distribution of the sintered samples were observed using a Quanta200 scanning electron microscope (FEI Company, Portland, OR, USA). Simple measurements of the pore structures and visible crystal sizes of the samples were taken from the SEM images using the ImageJ (1.80) image processing tool. The average size of ceramic crystal D_xrd_ (nm) and lattice strain *ε* (%) was determined using the Williamson–Hall method, which is based on the XRD pattern data of ceramic materials analyzed with Jade 9.0 software.

## 3. Results and Discussion

### 3.1. Analysis of the Orthogonal Experiment Results

Water absorption rate and modulus of rupture data gathered for ceramic tile samples prepared using the CaO-SiO_2_-MgO system and for samples prepared using steel slag and waste clay bricks as the primary raw materials are presented in Table 8. The table demonstrates that all the samples except those in the Zj-1 and Zj-9 groups met the requirements of the ISO 10545-3 [44] standard and ISO 10545-4 [45] standard, which stipulate that the modulus of rupture must be greater than or equal to 35 MPa. Notably, although the samples in the Zj-5 and Zj-6 groups had significantly higher fracture moduli than that required by the national standard, their water absorption rates exceeded the 3% limit. This result further confirms that adding steel slag positively affects the mechanical properties of ceramic tiles fabricated using the CaO-MgO-SiO_2_ system, which is consistent with results previously obtained by Zhao et al. [34] After conducting the range analysis, the corresponding results were derived from the data presented in Table 8 and subsequently documented in Table 9 and Table 10. In this study, ki represents the aggregate of the test results corresponding to level i under the factor represented in a given column of the table, while the R-value quantifies the magnitude of the impact exerted by the factor on the results. These tables show that temperature had the most significant influence on the water absorption rate, followed by the WCB content and finally the SS content. In contrast, for the modulus of rupture, the temperature still exerted the most significant influence, but it was followed by the SS content and finally the WCB content.

**Figure 5:**
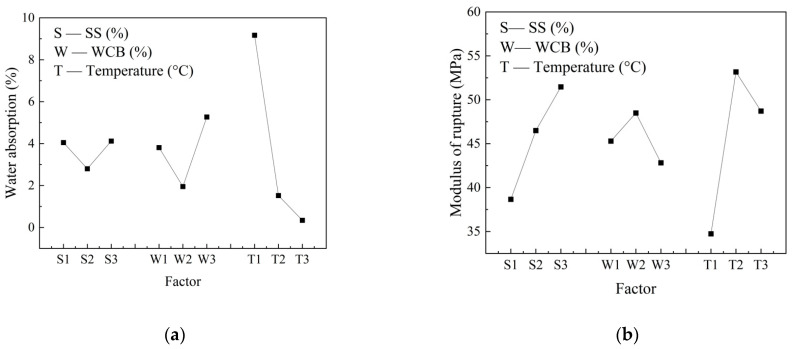
Visual analysis chart: (**a**) the modulus of rupture and (**b**) the water absorption rate.

Figure 5 illustrates the visual analysis of water absorption and modulus of rupture, with the x-axis representing different levels of each factor i and the y-axis representing the k*i*naverage value. By observing Figure 5, we can gain a more intuitive understanding of how different levels of each factor influence the experimental indexes and select appropriate levels to achieve optimal results. Three primary conclusions were inferred from the results presented in the figure. First, the water absorption rate initially decreased and then increased as the steel slag content increased, and reached its optimum value when steel slag comprised 40% of the total material. Meanwhile, the modulus of rupture continued to increase as the steel slag content increased, and reached its maximum value when steel slag comprised 45% of the total material. Second, as the waste clay brick content increased, the water absorption rate followed a similar trend in that it first decreased and then increased. It reached its optimal value when waste clay bricks accounted for 35% of the total material. Additionally, the modulus of rupture initially increased and then decreased. It also reached its peak value when waste clay bricks comprised 35% of the total material. Third, as temperature rose, the water absorption rate continuously decreased until it reached its optimum value, which occurred at a temperature of 1200 °C. The modulus of rupture, however, first increased and then decreased, and it reached its peak when the temperature was 1190 °C. Therefore, the optimal formulations for the modulus of rupture and the water absorption rate are 45% SS, 35% WCB, and 25% talcum powder with a sintering temperature of 1190 °C and 40% SS, 35% WCB, and 25% talcum powder with a sintering temperature of 1200 °C, respectively.

The results of validation experiments for these optimal formulations and the formulations with the best performance from the orthogonal experiments are shown in Table 11. Notably, the Y-2 group exhibited the best results with respect to all properties. Its water absorption was 0.08%, and its modulus of rupture was 63.68 MPa. The experimental results for the Y-2 group demonstrated that using steel slag and waste clay bricks as primary raw materials enabled the production of environmentally friendly ceramic tiles with mechanical properties that surpassed the requirements set by traditional standards ISO 10545 [44,45] (modulus of rupture ≥ 35 MPa, water absorption rate ≤ 0.5%), exhibiting nearly twice the required strength. Additionally, the Y-2 formula included a remarkable 76% proportion of solid waste. This significant proportion can lead to the conservation of conventional raw materials and reduced production costs. In conjunction with the results from Zhao et al. [34,35], it was observed that the samples from the Y-2 group in this study exhibited a lower modulus of rupture than ceramic tiles containing pyroxene as the primary crystalline phase but a higher modulus of rupture than ceramic tiles with calcium feldspar as the principal crystalline phase. Furthermore, the samples in group Y-2 achieved sintering at a temperature of 1190 °C, which is below that required for ceramic tiles composed predominantly of tremolite and calcium feldspar. Notably, the Y-2 ceramic samples demonstrated an enhanced solid waste utilization efficiency through the incorporation of raw materials derived from waste clay bricks as substitutes for conventional clay-based constituents.

**Figure 6:**
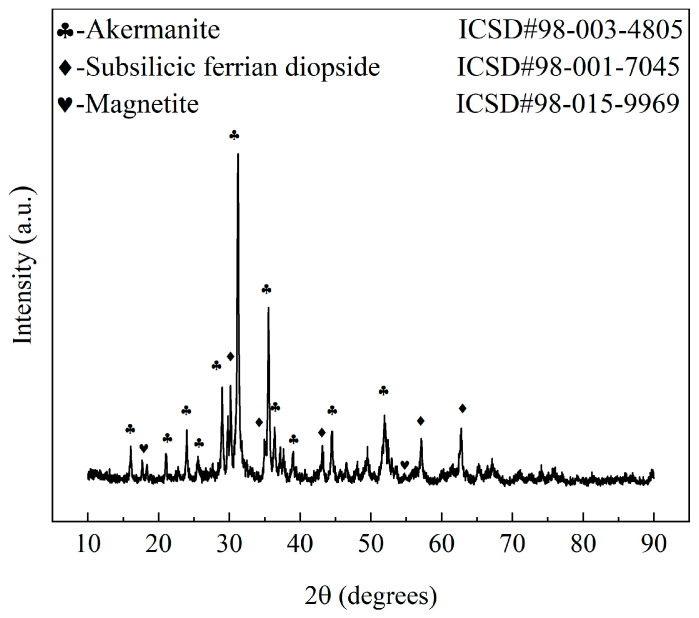
XRD pattern for a Y-2 sample.

The types, structures, and characteristics of the crystalline phases and the material microstructures determine the macroscopic properties of ceramic materials. The XRD pattern of the Y-2 sample, shown in Figure 6, indicates that the ceramic samples in this group belonged to the CaO-MgO-SiO_2_ system [35]. Unlike conventional ceramics, in which mullite is the primary stabilizing crystal phase [46], the crystal phases in these samples were more complex. Unlike the results of some previous studies [35,47], although these ceramic tiles belonged to the same CaO-MgO-SiO_2_ system, they predominantly exhibited stable phases of akermanite, subsilicic ferrian diopside, and magnetite. Notably, both the akermanite and subsilicic ferrian diopside were newly formed in the samples. In contrast, magnetite was the original crystalline phase in the raw material. The diffraction pattern of the raw material was eliminated, except for the magnetite, which suggests that the sample experienced comprehensive sintering, vitrification, and crystallization processes.

**Figure 7:**
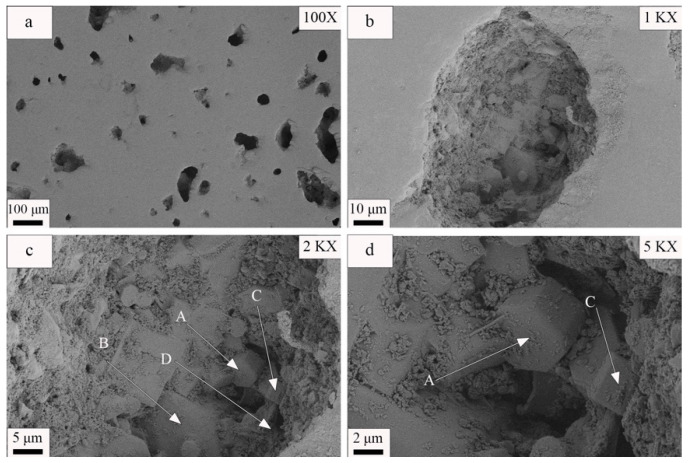
SEM images of a Y-2 sample at different magnifications. (**a**) SEM image of a Y-2 sample at 100× magnification. (**b**) SEM image of a Y-2 sample at 1000× magnification. (**c**) SEM image of a Y-2 sample at 2000× magnification. (**d**) SEM image of a Y-2 sample at 5000× magnification. (A and B correspond to akermanite, C indicates diopside, and D denotes magnetite).

The SEM microstructure of a Y-2 sample is illustrated in Figure 7. Figure 7a shows that the sample surface had nearly circular pores with diameters that ranged from 20 to 40 μm. This result indicates that the samples made using this formula had excellent densification degrees and low water absorption values. It is worth noting that only a few connected macropores with diameters greater than 100 μm were present, and they resulted from the merging of several tiny pores. This feature explains the superior mechanical properties of this sample. However, it is essential to recognize that pores can lead to cracking caused by their stress concentrations, which reduces the strength of the material. Therefore, controlling the number and sizes of the pores is crucial for effectively enhancing the mechanical properties and densification of ceramic materials [48]. The microstructure presented in Figure 7c reveals that the visible crystals of which it was composed had diameters that ranged from 2 to 6 μm. The crystals also had diverse morphologies, including short columnar, plate-like, and rhombic hexahedral structures. XRD analyses confirmed that the columnar and plate-like crystals with hexagonal bases (labeled A and B, respectively) corresponded to akermanite [43], the columnar crystals with irregular tetragonal bases corresponded to subsilicic ferrian diopside (labeled C) [49], and the rhombic hexahedral structures corresponded to magnetite crystals (labeled D) [50]. The crystals were densely packed and surrounded by a glassy substance. Columnar crystals occupied the pores formed by the plate-like crystals, while small grains filled the pores of the large grains. As a result, the ceramic materials exhibited excellent densification and mechanical strength properties.

**Figure 8:**
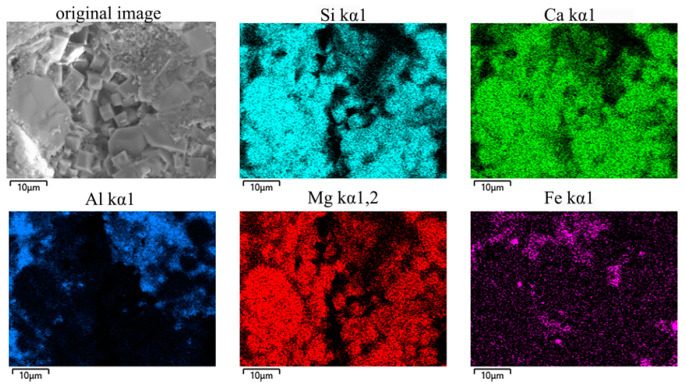
EDS analysis results for a Y-2 sample.

The distribution of each element in the Y-2 sample was determined through an energy dispersive spectroscopy (EDS) analysis. Figure 8 demonstrates that Si, Ca, and Mg were primarily concentrated in the akermanite and diopside phases, while Fe was mainly distributed in the glassy and magnetite phases. Only a trace amount of Fe was solidly dissolved into the diopside phase. Al was primarily distributed in the glassy phase. The distribution of each element was highly enriched, visibly apparent, and indicative of the high degree of crystallinity of the sample and of a significant vitrification effect, which indicates complete sintering.

### 3.2. Single-Factor Experiment Results and Analysis

#### 3.2.1. Effects of the Molding Pressure

**Figure 9:**
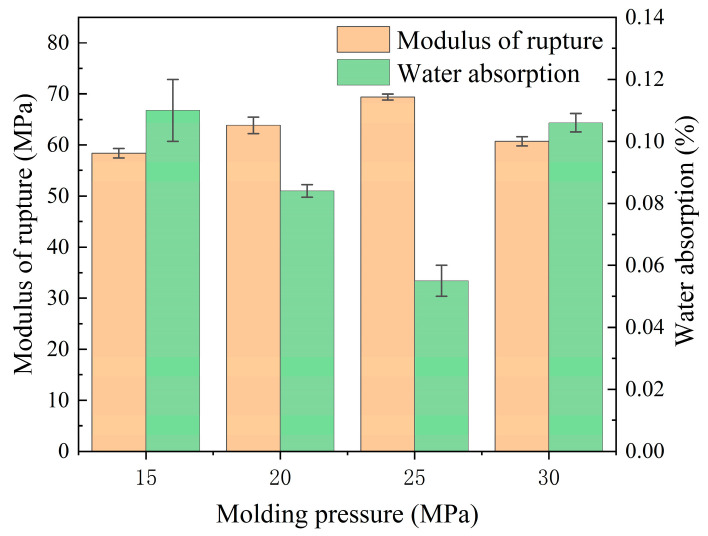
Water absorption and modulus of rupture results for ceramic tiles formed under different molding pressures.

The performance of ceramic tiles fabricated under pressures ranging from 15 to 30 MPa is illustrated in Figure 9. It can be observed that the modulus of rupture of ceramic tiles initially increased and then declined as the molding pressure increased from 15 to 30 MPa, while the water absorption rate exhibited an opposite trend. Notably, ceramic tiles prepared at a molding pressure of 25 MPa exhibited optimal performance, with a modulus of rupture of 69.21 MPa and a water absorption rate of 0.05. Furthermore, all sample groups demonstrated physical properties that exceeded ISO 10545-3 [44] standard and ISO 10545-4 [45] standard (i.e., modulus of rupture greater than 35 MPa and water absorption rate less than 0.5%). It is worth noting that, consistent with Erdogmus et al.’s findings [51], increasing the molding pressure within a certain range can enhance the physical properties of ceramic materials; however, exceeding a critical value will lead to performance deterioration.

The data presented in Table 12 indicate that the apparent porosity of the ceramic tile sample initially decreased and then increased with increasing molding pressure. Notably, at a molding pressure of 25 MPa, the minimum recorded value was only 0.086%. At the same time, both bulk density and linear shrinkage of the ceramic tile sample showed an initial increase followed by a subsequent decrease, reaching their maximum values at a molding pressure of 25 MPa. These findings suggest that increasing the molding pressure enhances internal densification within the ceramic material, resulting in reduced pore count, increased density, and elevated linear shrinkage [52]. However, excessive molding pressure can lead to decreased ceramic material densification.

**Figure 10:**
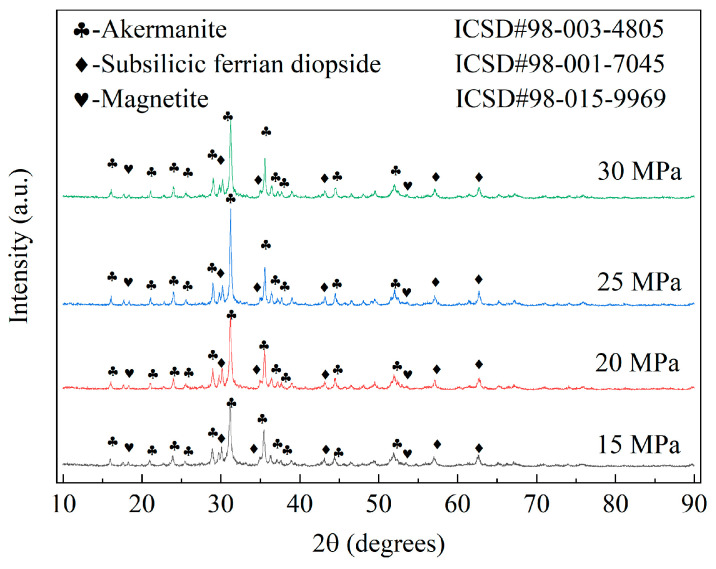
XRD patterns of samples created under different molding pressures.

Figure 10 depicts XRD patterns of the samples created using various molding pressures. The results reveal that the crystal compositions of the samples remained constant despite the differences in the molding pressure. However, the diffraction peak intensities of each phase were positively correlated with increases in the molding pressure. The optimal crystallinity of the material was observed when the molding pressure was 25 MPa, as indicated by the maximal diffraction peak intensity. Nevertheless, the intensities of the diffraction peaks began to decrease with further increases in the molding pressure. By examining the average grain size and lattice strain of ceramic crystals presented in Table 13, we can deduce the same law. With increasing molding pressure, there was an initial growth followed by a subsequent decline in crystal size, while the lattice strain exhibited an inverse trend. Notably, at a molding pressure of 25 MPa, the ceramic material attained its maximum average grain size alongside minimal lattice strain. Therefore, it can be inferred that an increase in molding pressure facilitates the development of ceramic crystals and mitigates lattice strain. However, excessive molding pressure hinders crystal growth, resulting in elevated lattice strain A similar phenomenon was observed during the study conducted by Pérez et al. [53], who investigated the impacts of the molding pressure on both the microstructure and process properties of stoneware tiles. Specifically, an increase in the molding pressure resulted in the formation of larger mullite particles while a consistent crystal morphology was maintained. These observations suggest that the molding pressure is quintessential to the sample crystallization process. Thus, it is imperative to maintain molding pressure conditions that will produce the optimal crystallinity.

**Figure 11:**
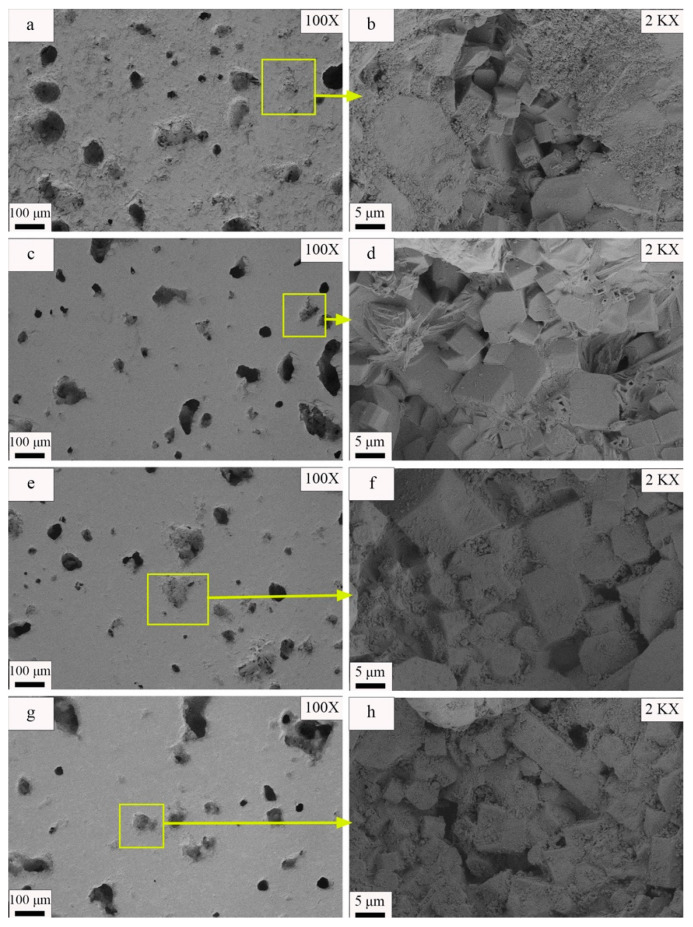
SEM images of samples at different molding pressures: (**a**) 15 MPa 100× SEM image, (**b**) 15 MPa 2000× SEM image, (**c**) 20 MPa 100× SEM image, (**d**) 20 MPa 2000× SEM image, (**e**) 25 MPa 100× SEM image, (**f**) 25 MPa 2000× SEM image, (**g**) 30 MPa 100× SEM image, and (**h**) 30 MPa 2000× SEM image.

**Figure 12:**
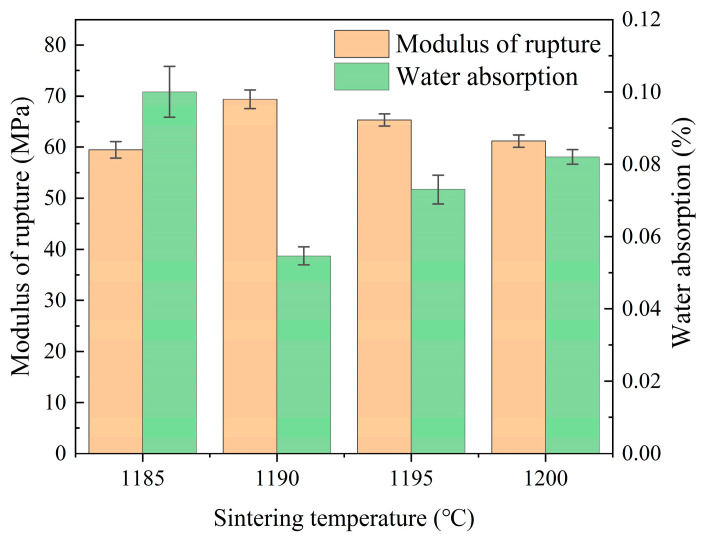
Water absorption and modulus of rupture results for samples created at different sintering temperatures.

Figure 11 present images of the samples formed under different molding pressures at magnifications of 100× and 2000×, respectively. The observable pores and visible grains in Figure 11 and Figure 12 were manually measured one-by-one using the dimensional measurement function in the ImageJ software. Subsequently, the average sizes of the pores and grains were calculated from these measurements. The results indicate gradual decreases in the sizes of the pores on the sample surface with increases in the molding pressure. In addition, Figure 11a,c,e,f demonstrate that the number of connected holes larger than 60 μm decreased and that the average pore size decreased from 46.27 μm to 35.75 μm as the molding pressure increased from 15 MPa to 25 MPa. However, if the molding pressure exceeded 25 MPa, the number of pores on the sample surface increased, the average pore size increased to 51.06 μm, and multiple linked macropores appeared on the sample surface. Figure 11b,d,f,h also reveal that the visible grain size variations within the samples followed the trends of the phase peaks in the XRD diffraction patterns. As molding pressure increased from 15 MPa to 25 MPa, the grain diameter increased from 4.03 μm to 6.02 μm. However, if the molding pressure increased to 30 MPa, the visible grain size decreased again to 4.23 μm.

Combining Figure 9, Figure 10 and Figure 11 and Table 12 and Table 13, the influence of molding pressure on the performance of ceramic tiles from the perspective of liquid-phase sintering was analyzed. During the process of liquid-phase sintering, an increase in temperature leads to the generation of a certain amount of liquid phase. The wetting effect of this liquid phase on solid particles results in the formation of capillaries between raw material particles [54]. These capillary forces establish connections among solid particles and facilitate their movement and rearrangement within the liquid medium, thereby achieving a denser structure [55]. Augmenting molding pressure reduces particle gaps, enhances capillary forces, and promotes particle rearrangement effects. Moreover, increasing molding pressure also decreases the required amount of molten liquid for filling voids while promoting particle rearrangement, ultimately leading to an increase in ceramic material density, a reduction in internal residual porosity, an increase in dimensional shrinkage, and an overall increase in density (refer to Figure 11 and Table 12) [56]. Furthermore, during mid-to-late stage sintering of ceramic materials, fine grain growth is primarily driven by grain boundary motion. When molding pressure is low and green body density is poor, internal gas pores impede grain boundary motion and reduce interfacial energy necessary for forward movement. Consequently, interfaces gradually flatten out and inhibit further grain growth. As molding pressure increases, hindrance caused by internal pores diminishes which facilitates more complete grain growth. However, exceeding the plastic deformation limit results in brittle fractures within green bodies, causing internal hardening defects and initially affecting particle rearrangement processes early on leading to a decreased level of densification inside materials but later impeding grain boundary motion, thereby suppressing finer grains’ growth (see Figure 10 diffraction peak intensity; Figure 11b,d,f,h; Table 13). Additionally, Yin et al. [57] have reported similar findings regarding the formation of internal cracks due to excessive compression deformation energy accumulation. The aforementioned conclusions are further supported by the data presented in Table 13. While refining grain size and increasing lattice strain can enhance the mechanical properties of specific alloy materials and single crystal materials, it is important to note that for the materials obtained in this experiment, excessively small grain size may be attributed to the incomplete solid-state reaction of ceramic materials and a low degree of crystallization within the ceramic material [58,59]. As a result, on a macroscopic scale, ceramics with smaller grain sizes exhibit lower fracture modulus, linear shrinkage, and density but demonstrate higher water absorption rate and apparent porosity. The aforementioned microlevel changes will have a direct impact on the macroscopic physical properties, as illustrated in Figure 9.

In summary, the molding pressure significantly influences the performance of such ceramic tiles prepared using the pressing molding method. Applying an appropriate molding pressure can facilitate close particle arrangement, decrease the number of internal pores, and enhance the densification and mechanical properties of the material. Nevertheless, applying excessive pressure can result in an overly dense material in which gas discharge and grain growth are impeded, thus ultimately negatively affecting the overall performance of the ceramic tiles. Hence, selecting an appropriate molding pressure during production is crucial to ensuring optimal ceramic tile performance. This conclusion is also applicable to other ceramic tile materials.

#### 3.2.2. Effects of the Sintering Temperature

The effects of different sintering temperatures on the properties of ceramic tiles were investigated while maintaining an optimal molding pressure at 25 MPa (see Section 3.2.1) and a soaking time of 90 min. The selected range for sintering temperature was from 118.5 °C to 1200 °C, as shown in Figure 12. The performance of each group of ceramic tiles was found to be significantly superior to the requirements set by the ISO 10545-3 [44] standard and ISO 10545-4 [45] standard (i.e., modulus of rupture greater than 35 MPa and water absorption rate less than 0.5%). The fracture modulus initially increased and then decreased with increasing sintering temperature, whereas the water absorption rate showed an opposite trend, reaching its highest value at 1190 °C (69.21 MPa and 0.05%, respectively). These results indicate that within a certain range, raising the sintering temperature contributes to enhancing both physical and mechanical properties of ceramic tiles; however, exceeding this range negatively impacts their performance. Furthermore, it is evident from Figure 12 that changes in sintering temperature have a more pronounced effect on the physical properties of ceramic products than variations in molding pressure—a finding consistent with Zhao et al. [34]’s previous research.

The data presented in Table 14 demonstrate that the apparent porosity of the ceramic tile sample initially decreased and subsequently increased with an increase in sintering temperature. Simultaneously, both bulk density and linear shrinkage of the ceramic tile sample exhibited an initial increase followed by a decrease, reaching their maximum at 1190 °C. These findings suggest that within a certain range, elevating the sintering temperature can effectively enhance the densification of the ceramic material, resulting in a reduction in pore quantity and an increase in density [60,61].

**Figure 13:**
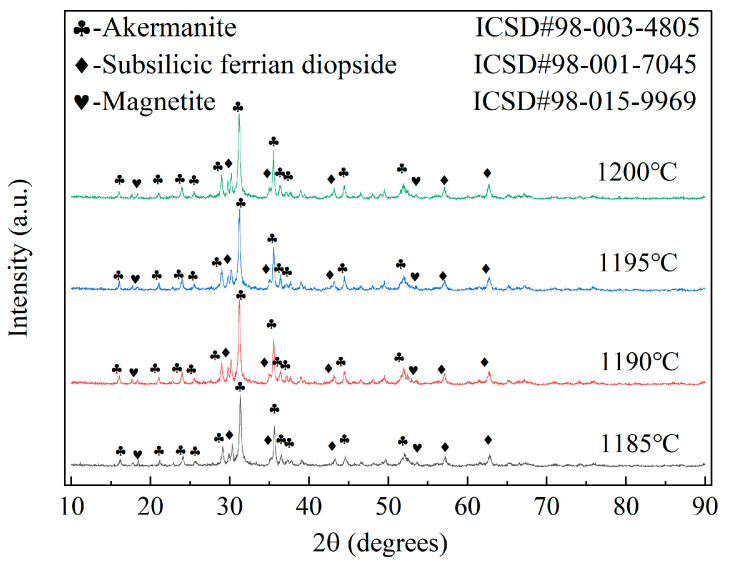
XRD patterns of samples produced with different sintering temperatures.

XRD patterns of samples fabricated at different sintering temperatures are presented in Figure 13. The results reveal that the crystalline phase compositions of the samples did not change as the sintering temperature increased. However, the intensities of the diffraction peaks of the phases identified in the XRD plots increased with increases in the sintering temperature [62]. This result suggests that the sintering temperature influenced the structural properties of the material but did not alter the composition. Simultaneously, the analysis of average grain size and lattice strain of ceramic crystals in Table 15 reveals a consistent trend (an initial increase followed by a decrease in crystal size with increasing sintering temperature), while lattice strain exhibited the opposite behavior. The ceramic material demonstrated its maximum average grain size and minimum lattice strain at a sintering temperature of 1190 °C. Therefore, it can be inferred that raising the sintering temperature promotes the growth of ceramic crystals and alleviates lattice strain. However, beyond a certain range, excessively high sintering temperatures hinder crystal growth, resulting in increased lattice strain.

**Figure 14:**
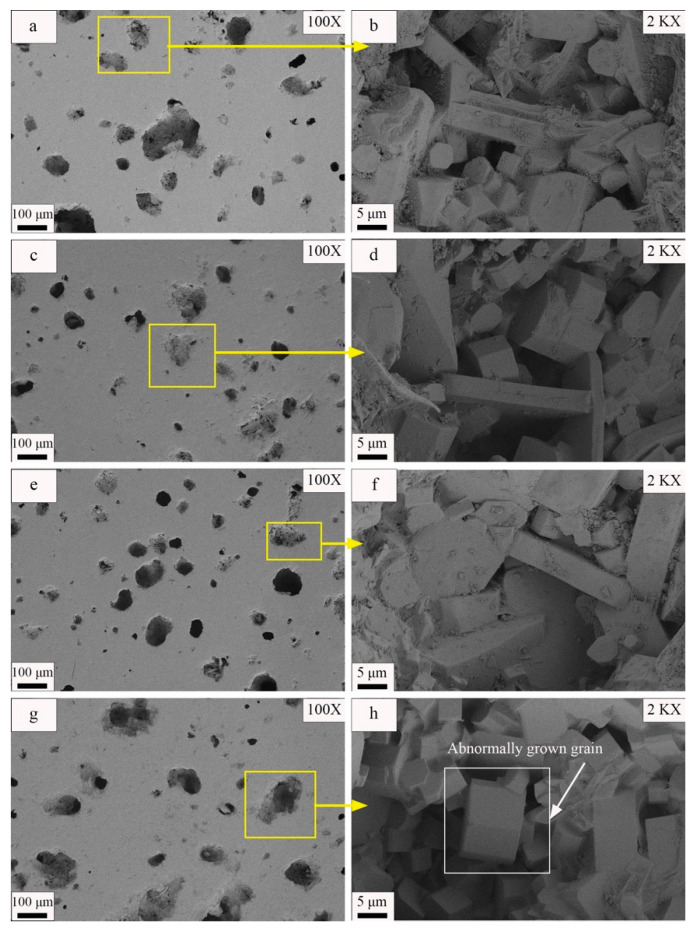
SEM images of samples under different sintering temperatures: (**a**) 1185 °C 100× SEM image, (**b**) 1185 °C 2000× SEM image, (**c**) 1190 °C 100× SEM image, (**d**) 1190 °C 2000× SEM image, (**e**) 1195 °C 100× SEM image, (**f**) 1195 °C 2000× SEM image, (**g**) 1200 °C 100× SEM image, and (**h**) 1200 °C 2000× SEM image.

Figure 14 depicts the microstructures of samples sintered at various temperatures. Observations of the 100× magnified SEM images (Figure 14a,c,e,g) indicate that the shapes and distributions of the pores on the sample surface changed with the sintering temperature. For a sintering temperature of 1185 °C, numerous irregularly shaped pores with an average diameter of 56 μm and several large pores with diameters exceeding 100 μm were present on the sample surface. An increase in the sintering temperature to 1190 °C caused the pores on the sample surface to become more uniformly distributed and to be rounder in shape. In addition, the average pore size decreased to 35.76 μm. However, as the sintering temperature continued to increase, the number of pores on the sample surface increased and the pores grew larger. For a sintering temperature of 1195 °C, the average pore diameter was 38.63 μm, while it was 54.66 μm for a sintering temperature of 1200 °C. At 1200 °C, the surface contained several pores with diameters larger than 80 μm; additionally, the hole distribution was concentrated, and neighboring small holes tended to merge and form large holes. The SEM images gathered at a 2000× magnification (Figure 14b,d,f,h) enabled observations of the visible crystal size variations within the material as the sintering temperature changed. Gradual increases in the visible grain size were evident as the sintering temperature increased. For instance, the average grain diameter increased from 6.9 μm to 9.86 μm when the sintering temperature increased from 1185 °C to 1200 °C.

Furthermore, an examination of the SEM image of the sample sintered at 1200 °C (Figure 14h) indicates that a few grains exhibited an abnormal growth pattern, having sizes significantly larger than those of the neighboring grains. Analysis of Figure 14 demonstrates that the material microstructure changed considerably with the sintering temperature. At lower temperatures, the surface of the sample contained many irregularly shaped pores, while at higher temperatures, the pores became rounder and more uniformly distributed. Moreover, elevated temperatures could potentially lead to pore wall fracture, thereby resulting in reduced porosity and enhanced tile densification [63]. Simultaneously, the grain size gradually increased with increases in the sintering temperature. However, abnormal grain growth occurred at the highest temperature (1200 °C), as shown in Figure 14h. These changes can significantly impact the physical properties of the material. Thus, further investigation is necessary to enable better understanding and control of the sintering process.

The liquid phase is pivotal to the sintering process of ceramic materials; it not only facilitates pore filling, which causes densification, but also effectively promotes crystal growth [64]. From the perspective of liquid-phase sintering, analysis of Figure 12, Figure 13 and Figure 14 and Table 14 and Table 15 reveals that a limited generation of the liquid phase occurred within the ceramic raw materials at low sintering temperatures. Consequently, the original material particles were completely enveloped, and filling the interstitial spaces between particles posed challenges. In this scenario, particle rearrangement ensued but failed to fully eliminate porosity, resulting in elevated apparent porosity and density in ceramic tiles exhibiting smaller linear shrinkage (refer to Figure 14 and Table 14). As the sintering temperature increased, a greater amount of liquid phase was generated within the ceramic material. During this stage, there occurred a mass flow transfer and dissolution/precipitation process inside the ceramic material, which involved both particle rearrangement and substantial pore filling. Consequently, densification was enhanced, apparent porosity was reduced, and density and linear shrinkage were increased. An expression for the sintering rate, which was derived from the viscous-flow mass transfer process during sintering, is presented in Equation (8):

(8) $$d\theta /dt=\frac{3}{2}\times \frac{\gamma }{r\eta }\left(1-\theta \right)$$

where *θ* represents the relative density, *r* is the particle radius, *γ* is the liquid–gas surface tension, *η* is the viscosity of liquid, and *t* is the sintering time.

From the formula, it can be observed that as the temperature increased, viscosity decreased, and the volume of the liquid phase increased. This resulted in a rapid increase in ceramic densification rate while also promoting grain growth through the liquid phase [65]. Sintering is the process of densification in a material during which the pores gradually shrink and internal pressure increases. When the pressure inside the pores reaches equilibrium with the sintering force, sintering stops. However, excessive sintering temperature can cause pore expansion instead of the desired contraction due to increased pressure within them. This phenomenon hinders densification and may result in decreased performance of ceramic materials (Figure 14g and Table 15). Moreover, when the sintering temperature exceeds a certain range, an excessive amount of liquid phase forms within the material. The particles lose their ability to support sintering and become susceptible to high-temperature creep deformation. Consequently, material deformation occurs and persists as temperature rises. At this stage, the significant presence of the liquid phase also promotes secondary recrystallization characterized by abnormal growth of individual grains leading to the formation of a few large grains (Figure 14h). These larger grains are prone to hidden cracks internally due to stress from surrounding grain boundaries [66]. The phenomena observed in Table 15 strongly support the analysis and discussion mentioned above. The microlevel changes had a simultaneous impact on the macrolevel performance of water absorption and modulus of rupture. As shown in Figure 12, an increase in sintering temperature initially optimized the water absorption and modulus of rupture of ceramic tiles; however, they deteriorated rapidly beyond 1190 °C. Therefore, choosing an appropriate sintering temperature is essential for obtaining ceramic tiles that perform excellently. Using an appropriate sintering temperature ensures that the necessary amount of liquid is maintained, thereby promoting grain growth and improving the various properties of the material while simultaneously achieving optimal densification with no material deformation or mechanical property deterioration.

Zhao et al. [35], Tabit et al. [37], and Wang et al. [67] found that incorporating steel slag can significantly decrease the sintering temperature required for ceramic materials; this finding aligns with the perspective presented in this study. This phenomenon may be attributed to the introduction of alkali metal elements, such as CaO, Fe_2_O_3_, and TiO_2_, into the raw materials, which causes a reduction in the eutectic temperature within the system. Consequently, ceramic tile samples prepared under this method require significantly lower sintering temperatures than those prepared using traditional methods (which typically employ temperatures greater than 1220 °C), which results in energy consumption reductions. However, the lowered eutectic point causes a narrower sintering range for the ceramic tiles, which may result in excessive liquid formation at high temperatures, which in turn can cause product deformation and quality issues. Therefore, it is imperative to control the proportion of alkali metal oxides in the raw materials and carefully select appropriate sintering conditions when designing ceramics formulas.

#### 3.2.3. Effects of the Soaking Time

**Figure 15:**
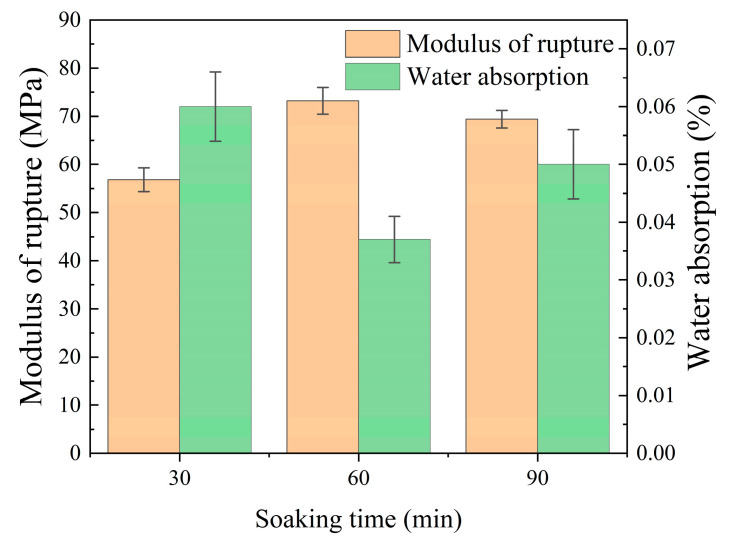
Water absorption and modulus of rupture results for samples created with different soaking times.

The influence of soaking time on the water absorption and modulus of rupture of the ceramic tiles, which were prepared in this study at the optimum molding pressure of 25 MPa and sintered at the optimum temperature of 1190 °C (as determined in Section 3.2.2), is illustrated in Figure 15. The results reveal that, as the soaking time increased from 30 to 90 min, the water absorption initially decreased and then increased, while the modulus of rupture exhibited an opposite trend. However, when the soaking time was 60 min, the water absorption reached its lowest value of 0.04%, while the modulus of rupture reached its maximum value of 73.2 MPa. Additionally, the physical and mechanical properties of each group surpassed the limits prescribed by ISO 10545-3 [44] standard and ISO 10545-4 [45] standard. These results demonstrate the significant impact that the soaking time has on ceramic tile performance.

The data presented in Table 16 demonstrate that the apparent porosity of the ceramic tile sample initially decreased and then increased with increasing soaking time. Specifically, when the soaking time was 60 min, it reached its minimum value. Simultaneously, both bulk density and linear shrinkage exhibited an initial increase followed by a decrease, reaching their maximum values at a soaking time of 60 min. These findings indicate that within a certain range, prolonging the holding time can effectively enhance densification of the ceramic material, resulting in reduced pore quantity and increased density. However, excessive holding times lead to over-densification of the ceramic material, causing an increase in apparent porosity as well as surface density while decreasing linear shrinkage [68,69].

**Figure 16:**
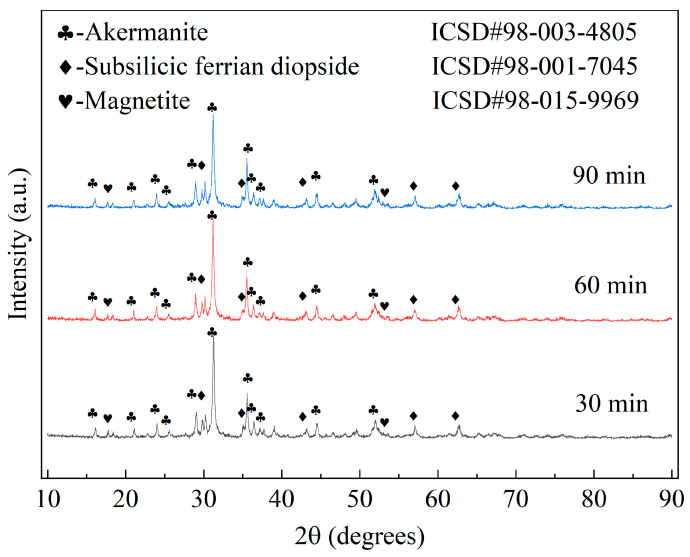
XRD patterns of samples produced using different soaking times.

XRD patterns presented in Figure 16 illustrate the crystal phase compositions and diffraction peak intensities of the samples fabricated using various soaking times. It is noteworthy that the crystal phase composition remained unchanged with increasing soaking time, and there was no significant alteration in the diffraction peak intensity of each phase. However, upon examination of Table 15, it is evident that the average grain size of ceramic crystals initially increased and subsequently decreased with increasing holding time, while the lattice strain exhibited an opposite trend. The largest average grain size of the ceramic material and the smallest lattice strain were observed at a holding time of 60 min. Therefore, it can be inferred that prolonged soaking time promotes the development of ceramic crystals while reducing lattice strain. Excessive soaking time hinders crystal growth, resulting in increased lattice strain and decreased crystal size.

**Figure 17:**
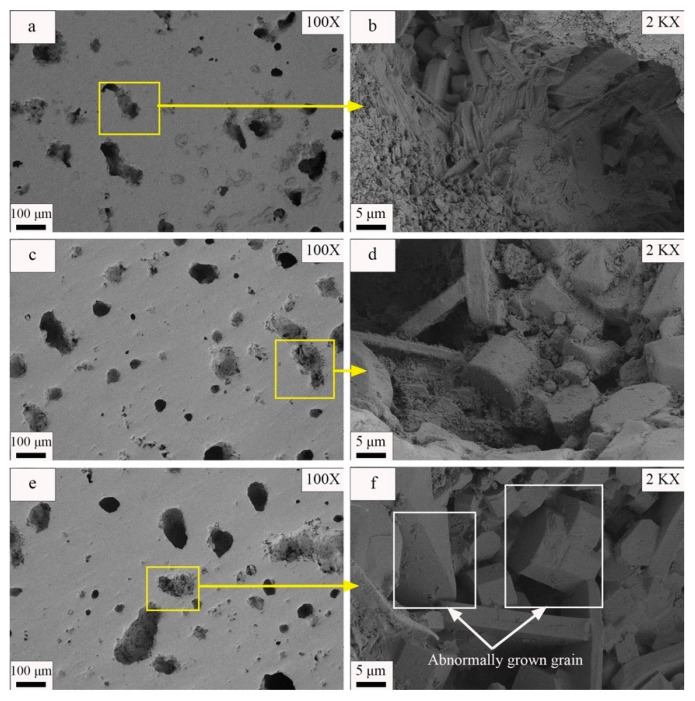
SEM images of samples at different soaking times. (**a**) Soaking time of 30 min 100× SEM image, (**b**) soaking time of 30 min 2000× SEM image, (**c**) soaking time of 60 min 100× SEM image, (**d**) soaking time of 60 min 2000× SEM image, (**e**) soaking time of 90 min 100× SEM image, and (**f**) soaking time of 90 min 2000× SEM image.

The SEM images presented in Figure 17 provide detailed insight into the surface morphologies and internal structures of the samples prepared with different soaking times. A close examination of the images reveals that, when the soaking time was 30 min, the sample surface contained numerous pores. These pores were formed by the coalescence of several tiny pores, which ultimately resulted in connected macropores with pore diameters that ranged from 80 to 140 μm. As a result, the samples exhibited poor densification. However, when the soaking time increased to 60 min, the pores on the sample surface decreased in size and acquired more circular shapes; only a few pores were left that had diameters in excess of 100 μm. Therefore, these samples possessed good densification. When the soaking time increased to 90 min, however, the sizes of the surface pores increased and several large elongated pores appeared. These pores had diameters greater than 100 μm, which resulted in poor densification of the ceramic material. The images presented in Figure 17b,d,e also reveal that the visible grain sizes within the material increased with increases in the soaking time. However, when the soaking time was 90 min, secondary crystallization occurred within the samples, which caused abnormal growth of individual grains inside the material. This abnormal growth further resulted in deterioration of the mechanical properties of the material and increased porosity.

In high-temperature conditions, the duration of soaking time is a key factor that affects the densification and grain growth processes of ceramic samples. This is because the sintering process of materials is characterized by the following two main diffusion mechanisms: volume diffusion and surface diffusion. The low-temperature stage is mainly influenced by surface diffusion [70]. Although it can alter the morphology of surface pores, its contribution to material densification is not significant. Densification primarily relies on volume diffusion occurring in the high-temperature stage [71]. Therefore, the duration of soaking time has an important impact on the volume diffusion taking place at high temperatures.

Based on the findings depicted in Figure 15, Figure 16 and Figure 17 and Table 16 and Table 17, it is evident that the duration of soaking time significantly affects the characteristics of ceramic tile materials. While prolonging the soaking time does not alter the phase composition within the ceramic material, it enhances volume diffusion at high temperatures during longer durations of soaking time. Therefore, increasing the soaking time from 30 min to 60 min will facilitate the densification of ceramic tiles, resulting in enhanced density, linear shrinkage, and grain size while reducing apparent porosity. However, prolonged soaking time can result in the non-uniform growth of secondary grains within the ceramic material, which leads to a reduction in density. The data presented in Table 17 provide strong support for the aforementioned inference. This phenomenon was also observed in the study conducted by Manan et al. [72] investigating the impact of holding time on the densification performance of Sr_0.5_Ca_0.5_La_4_Ti_5_O_17_ ceramics (as depicted in Figure 17f). These microscopic changes manifest as variations in water absorption and modulus of rupture of the ceramic material demonstrated macroscopically in Figure 15.

For most ceramic materials, precise control of the soaking time is crucial when preparing ceramic samples with optimal densities and excellent properties. Utilization of this practice can ensure higher densities, thereby making the material denser and more complex, and uniformly sized grains, which significantly enhance the overall properties of the material. Therefore, it is important to employ an appropriate soaking time in order to achieve optimal ceramic samples that feature high densities and superior properties.

#### 3.2.4. Economic Analysis

The results presented in Table 18 demonstrate the disparities in performance between the ceramic samples obtained in this experiment and various commercially available architectural ceramic brick samples. It is evident that the rupture modulus of the ceramic material derived from this study surpasses that of multiple groups of commercial ceramic tiles while exhibiting the lowest water absorption rate among all four groups.

The primary cost structure of traditional ceramic tiles includes raw material costs, labor expenses, energy expenditures, and other miscellaneous outlays such as transportation, packaging, and storage. Raw material costs account for approximately one-third of the total cost of ceramic tiles, while labor costs make up around one-fourth to one-third. Current energy costs represent about one-fifth of the overall expenditure, with other expenses constituting roughly one-tenth to one-twentieth. Traditional ceramic tiles mainly consist of 70% porcelain clay, 20% porcelain stone, and 10% quartz.

Considering fixed costs excluding raw materials, we will explore the impact on production costs by utilizing steel slag and waste clay in ceramics manufacturing. Currently, in China’s market, porcelain clay is priced at CNY 1000 per ton; porcelain stone at CNY 1500 per ton; quartz at CNY 400 per ton; and industrial talcum at CNY 950 per ton. According to the traditional ceramics formulae mentioned above, prices result in a raw material cost of CNY 1040 per ton.

However, since steel slag and waste clay are heavily accumulated solid wastes, only the auxiliary raw material talcum’s price is considered; hereafter, this experiment’s optimal formula yields a mere price of CNY 226 for each metric ton of raw materials, reducing raw material expenses by an impressive margin, namely by 78%, and, consequently, leading to an overall reduction in ceramics’ production costs amounting to 26%.

## 4. Conclusions

This study focused on the preparation of akermanite ceramic tiles that utilized steel slag and waste clay bricks as primary raw materials. Using an orthogonal experimental design, the optimal formula for these ceramic tiles was identified and the influence of various technological parameters on the sintering behavior and material properties was extensively discussed. This research produced seven key conclusions:

(1) Using an L_9_ (3^3^) orthogonal experiment and range analyses, an optimal formula was obtained for a ceramic tile material that used akermanite as the primary crystalline phase. The formula consisted of 45% steel slag, 35% waste clay brick powder, and 25% talc; thus, 76% of its composition was solid waste. The material was sintered at a temperature of 1190 °C. The ceramic tile samples produced using this formula exhibited a modulus of rupture of 63.68 MPa and a water absorption rate of 0.08%; thus, they satisfied the requirements of the ISO 10545-3 standard and ISO 10545-4 standard (which specified that the modulus of rupture should be greater than or equal to 35 MPa, while the water absorption rate should be less than or equal to 0.5%).
(2) XRD patterns obtained for the samples demonstrated that akermanite, diopside, and magnetite were the primary crystalline phases present in the samples fabricated with this particular composition.
(3) The ideal process parameters to use when producing ceramic samples with this composition were determined from a single-factor experiment. They included a molding pressure of 25 MPa, a sintering temperature of 1190 °C, and a soaking time of 60 min. The ceramic tiles, produced with the optimal composition and process parameters, had a modulus of rupture of 73.2 MPa and a water absorption rate of only 0.04%, surpassing the requirements of the ISO 10545 standard.
(4) The appropriate increase in molding pressure promoted particle rearrangement, reduced the required melt volume, improved ceramic density, enhanced crystal development, and optimized material performance. Excessive molding pressure led to hardening defects in the green body, resulting in decreased density, incomplete crystal development, and deteriorated material performance. In this experiment, the fracture modulus at the optimal molding pressure was 69.21 MPa, with a water absorption rate of 0.05.
(5) Appropriate increases in sintering temperature facilitated the formation of a liquid phase, promoting ceramic densification, crystal growth, and optimization of material properties. However, excessive sintering temperature could lead to an excess of liquid phase formation, causing ceramic deformation, abnormal grain growth, and deterioration of material properties. In this experiment, the optimal sintering temperature was determined as 1190 °C, resulting in a modulus of rupture of 69.21 MPa and a water absorption of 0.05.
(6) Appropriate increases in soaking time promoted volume expansion, densification, crystal development, and optimization of ceramic material performance at high temperatures. Excessive soaking time led to non-uniform growth of secondary grains in ceramics, resulting in the deterioration of material performance. In this experiment, the fracture modulus obtained at the optimal sintering temperature of 1190 °C was 73.2 MPa, with a water absorption of 0.04%.
(7) The ceramic tiles exhibited a water absorption rate of only 0.04% and possessed a fracture modulus of 73.2 MPa, surpassing that of commercially available samples. Furthermore, the cost per metric ton of raw materials was only CNY 226, representing a remarkable reduction by 78% compared with traditional materials.

## Figures and Tables

**Table 1:** Chemical compositions of the raw materials (wt.%).

	SiO_2_	Al_2_O_3_	Fe_2_O_3_	MgO	CaO	Na_2_O	K_2_O	MnO	P_2_O_5_	TiO_2_	LOI
**SS**	14.53	3.40	20.49	5.41	34.92	0.21	0.07	6.00	1.98	1.39	1.3
**WCB**	47.03	12.97	5.72	2.32	8.78	1.61	2.27	0.13	0.19	0.72	2.1
**Talc**	64.07	0.55	0.08	31.7	0.32	-	-	-	-	-	4.8

**Table 2:** Values for all levels of each factor in the orthogonal experiment.

Level	Experimental Factor		
	SS (%)	WCB (%)	Temperature (°C)
1	35	40	1180
2	40	35	1190
3	45	30	1200

**Table 3:** L_9_ (3^3^) orthogonal experiment design.

No.	Experimental Factor		
	SS (%)	WCB (%)	Temperature (°C)
Zj-1	35	40	1180
Zj-2	35	35	1190
Zj-3	35	30	1200
Zj-4	40	40	1200
Zj-5	40	35	1180
Zj-6	40	30	1190
Zj-7	45	40	1190
Zj-8	45	35	1200
Zj-9	45	30	1180

**Table 4:** Optimized formulations obtained from the orthogonal experiment.

No.	SS (%)	WCB (%)	Talc (%)	Temperature (°C)
Y-1	40	35	25	1200
Y-2	45	35	25	1190

**Table 5:** Single factor experiment of optimized formula (molding pressure).

No.	Molding Pressure (MPa)	Temperature (°C)	Soaking Time (min)
YY-1	15	1190	90
YY-2	20	1190	90
YY-3	25	1190	90
YY-4	30	1190	90

**Table 6:** Single factor experiment of optimized formula (temperature).

No.	Molding Pressure (MPa)	Temperature (°C)	Soaking Time (min)
YW-1	25	1185	90
YW-2	25	1190	90
YW-3	25	1195	90
YW-4	25	1200	90

**Table 7:** Single factor experiment of optimized formula (soaking time).

No.	Molding Pressure (MPa)	Temperature (°C)	Soaking Time (min)
YB-1	25	1190	30
YB-2	25	1190	60
YB-4	25	1190	90

**Table 8:** Results of the orthogonal experiment.

No.	Modulus of Rupture (MPa)	Water Absorption (%)
Zj-1	31.60	10.81
Zj-2	42.27	1.01
Zj-3	42.12	0.33
Zj-4	42.43	0.44
Zj-5	41.63	4.58
Zj-6	55.40	3.37
Zj-7	61.84	0.19
Zj-8	61.58	0.25
Zj-9	30.95	12.12

**Table 9:** Water absorption rate range analysis results.

No.	Experimental Factor		
	SS (%)	WCB (%)	Temperature (°C)
k*i*1	12.15	11.44	27.51
k*i*2	8.39	5.84	4.57
k*i*3	12.56	15.82	1.02
k*i*1average	4.05	3.81	9.17
k*i*2average	2.80	1.95	1.52
k*i*3average	4.12	5.27	0.34
R*i*	1.39	3.33	7.65

Note: k*i*n is the sum of the water absorption rate values; k*i*naverage is the average water absorption rate value; R*i* = max (k*i*naverage)-min(k*i*naverage); k*i*naverage = (k*i*1 + k*i*2 + k*i*3)/3; *i* = SS, WCB, temperature; n = 1, 2, 3.

**Table 10:** Modulus of rupture range analysis results.

No.	Experimental Factor		
	SS (%)	WCB (%)	Temperature (°C)
k1	115.99	135.87	104.18
k2	139.46	145.48	159.51
k3	154.37	128.47	146.13
k*i*1average	38.66	45.29	34.73
k*i*2average	46.49	48.49	53.17
k*i*3average	51.46	42.82	48.71
R*i*	12.79	5.67	18.44

Note: k*i*n is the sum of the modulus of rupture values; k*i*naverage is the average modulus of rupture value; R*i* = max (k*i*naverage)-min(k*i*naverage); k*i*naverage = (k*i*1 + k*i*2 + k*i*3)/3; *i* = SS, WCB, temperature; n = 1, 2, 3.

**Table 11:** Experimental results for the optimized formulations.

No.	Modulus of Rupture (MPa)	Water Absorption (%)
Y-1	35.80	0.10
Y-2	63.68	0.08

**Table 12:** Variation of molding pressures on the physical properties of samples.

	Apparent Porosity (%)	Bulk Density (g/m^3^)	Linear Shrinkage (%)
YY-1	0.171	2.56	12.9
YY-2	0.130	2.61	13.5
YY-3	0.086	2.78	14.6
YY-4	0.170	2.56	12.8

**Table 13:** Crystal parameters of samples under different molding pressures.

	Crystal Size (nm)	Lattice Strain (%)
YY-1	43.6	0.109
YY-2	52.8	0.076
YY-3	60.6	0.069
YY-4	46.5	0.141

Note: the sintering temperature for each experimental group in the table was set at 1190 °C.

**Table 14:** Variation of sintering temperatures on the physical properties of samples.

	Apparent Porosity (%)	Bulk Density (g/m^3^)	Linear Shrinkage (%)
YW-1	0.155	2.62	13.5
YW-2	0.086	2.78	14.6
YW-3	0.114	2.68	13.8
YW-4	0.129	2.64	13.6

**Table 15:** Crystal parameters of samples at different sintering temperatures.

	Crystal Size (nm)	Lattice Strain (%)
YW-1	36.5	0.046
YW-2	60.6	0.069
YW-3	43.8	0.108
YW-4	37.1	0.124

Note: sintering temperatures of each experimental group in the table are from YW1-YW4, which are 1185 °C, 1190 °C, 1195 °C, and 1200 °C, respectively.

**Table 16:** Variation of soaking times on the physical properties of samples.

	Apparent Porosity (%)	Bulk Density (g/m^3^)	Linear Shrinkage (%)
YB-1	0.101	2.69	13.8
YB-2	0.058	2.82	14.9
YB-3	0.086	2.78	14.6

**Table 17:** Crystal parameters of samples under different soaking times.

	Crystal Size (nm)	Lattice Strain (%)
YB-1	42.9	0.062
YB-2	47.8	0.113
YB-3	60.6	0.069

Note: sintering temperature for each experimental group in the table was set at 1190 °C.

**Table 18:** Performance comparison between experimental and commercial samples.

	ISO Standard	Experimental Sample	Commercial Sample 1	Commercial Sample 2	Commercial Sample 3
Water absorption (%)	E ≥ 0.5	0.04	0.33	0.05	0.02
Modulus of rupture (MPa)	R ≥ 35	73.2	38	47	49

## Data Availability

The raw and processed data required to reproduce these results are available upon reasonable request.
